# An Explorative Anatomical Study on Inter-Individual Variation of the Tibial Nerve and Landmarks for Perineural Anesthesia in Horses

**DOI:** 10.3390/ani14152161

**Published:** 2024-07-24

**Authors:** Margot De Schryver, Maarten Oosterlinck

**Affiliations:** Department of Large Animal Surgery, Anaesthesia and Orthopaedics, Faculty of Veterinary Medicine, Ghent University, 9820 Merelbeke, Belgium; margot.deschryver@ugent.be

**Keywords:** diagnostic anesthesia, lameness examination, landmarks, perineural anesthesia, tibial nerve, sports medicine

## Abstract

**Simple Summary:**

Perineural anesthesia of the tibial nerve can be performed ultrasound-guided or blindly, with the latter still being commonly used in equine practice due to practical constraints, despite its lower accuracy and hence, its common failure to achieve desensitization. This may be associated with anatomical variations or inadequate landmarks for injection. In this anatomical study on the course of the tibial nerve on paired cadaveric limbs, we found that problems with perineural anesthesia cannot simply be attributed to anatomical variations. The large diameter of the tibial nerve (6 ± 1 mm) and the substantial amount of perineural tissue may present specific challenges for achieving adequate desensitization. Regarding landmarks, this study confirms that perineural injection should be performed 10 cm proximal to the proximocranial aspect of the calcaneal tubercle and 11 mm cranial to the superficial digital flexor.

**Abstract:**

Perineural anesthesia of the tibial nerve can be performed ultrasound-guided or blindly, with the latter still being commonly used in equine practice due to practical constraints, despite its lower accuracy and hence, common failure to achieve desensitization. This may be associated with anatomical variations or inadequate landmarks for injection. To examine the course of the tibial nerve, document potential anatomical variations, and determine optimal landmarks for perineural injection, dissection was conducted along the medial aspect of the tibia in 10 paired cadaver hindlimbs. No anatomical variations of the tibial nerve were observed. Mean tibial nerve thickness was 6 ± 1 mm. The junction with the plantar nerves was located at a maximum of 85 mm and the junction with the medial cutaneous branch was at a maximum of 150 mm proximal to the proximal aspect of the calcaneal tubercle. The mean distance of the tibial nerve to the cranial border of the superficial digital flexor was 11 ± 6 mm. In conclusion, problems with perineural anesthesia of the tibial nerve cannot simply be attributed to anatomical variations. The thickness of the nerve and the amount of perineural tissue may present specific challenges for achieving adequate desensitization. Our results support the generally recommended site for tibial nerve perineural injection at 100 mm proximal to the calcaneal tubercle and 11 mm cranial to the superficial digital flexor.

## 1. Introduction

Perineural anesthesia of the tibial nerve can be used as a diagnostic technique during the lameness examination, mostly combined with perineural anesthesia of the fibular nerve for desensitization of the distal limb up to the tarsus. It can also be used for supplementary analgesia in surgical procedures as well as cases with chronic pain [1].

In several handbooks and publications, the technique has been described as a blind injection of 10–15 mL of local anesthetic solution with a 20–22 gauge needle, approximately 10 cm proximal to the calcaneal tubercle, in between the common calcaneal tendon (CCT) and the deep digital flexor (DDF), at a depth of 25–38 mm [1,2,3,4]. Dyson (1984) described the landmark for injection as a hand width above the calcaneal tubercle and at a depth of 1 cm [5]. Wheat and Jones (1981) stated that the tibial nerve is a large structure surrounded by a fascia, for which a large amount of anesthetic solution is required to achieve desensitization [6]. According to Bassage and Ross (2011), an alternative blind technique could involve inserting the needle between the CCT and DDF from the lateral aspect of the limb, despite the tibial nerve being located on the medial aspect of the limb [3]. It has been recommended that the response to tibial neural anesthesia should be assessed in 10 to 30 min, as the location and the size of the nerve may require a longer time for diffusion of the anesthetic [3,7].

As an alternative to blind injection, ultrasound guidance can be useful to facilitate perineural injection of the tibial nerve. Van der Laan et al. [8] researched the accuracy of a blind versus an ultrasound-guided technique on 42 paired cadaveric limbs from 21 horses using 1 mL methylene blue, and they reported that the success rate of nerve staining was significantly higher using the ultrasound-guided technique (85.7%) than with the blind technique (47.6%; *p* = 0.02; OR 6.6; 95% CI, 1.5–29.4). However, in that study, a single ultrasound-guided injection did not consistently result in nerve staining [8]. Denoix et al. [7] reported an ultrasound-guided injection technique in which the needle was first inserted caudally to the probe and directed to the caudal aspect of the nerve. Half of the volume of anesthetic solution was injected (5–8 mL) at this site and subsequently, a second injection was made similarly, cranial to the probe [7].

An in vivo comparison of the blind and ultrasound-guided techniques was reported by Bellitto et al. (2024) [9]. They found that the ultrasound-guided technique with an injection cranial and caudal to the nerve was superior to the blind technique, as in the former technique, desensitization of the heel bulbs was achieved in all horses after a period of 30–35 min. However, two operators were required for the technique and it took significantly longer to perform than the blind technique [9].

Notwithstanding the superior accuracy of injection with ultrasound guidance, the blind technique is still used in equine practice due to practical constraints, resulting in common failure to achieve desensitization. Therefore, the aim of this explorative study was to examine the course of the tibial nerve, to document potential anatomical variations, and to determine optimal landmarks for perineural injection on paired cadaveric limbs.

## 2. Materials and Methods

Ten intact hindlimbs from five adult warmblood horses euthanized for reasons unrelated to this study were used. Clinical records, breed and height at the withers of the horses were unknown. The paired hind limbs were disarticulated at the coxofemoral joint and dissected along the medial aspect of the tibia.

### 2.1. Anatomical Dissection

The hind limbs were dissected along the medial aspect of the tibia using a long curvilinear incision starting just proximal to the femoral head, to the distal aspect of the tarsus. The deep crural fascia was dissected and perineural fat and connective tissue were separated. The tibial nerve, along with its plantar branches and medial cutaneous branch, were identified. In certain specimens, the saphenous vein was either partially or entirely excised to facilitate visualization of the tibial nerve.

### 2.2. Measurements

**Figure 1:**
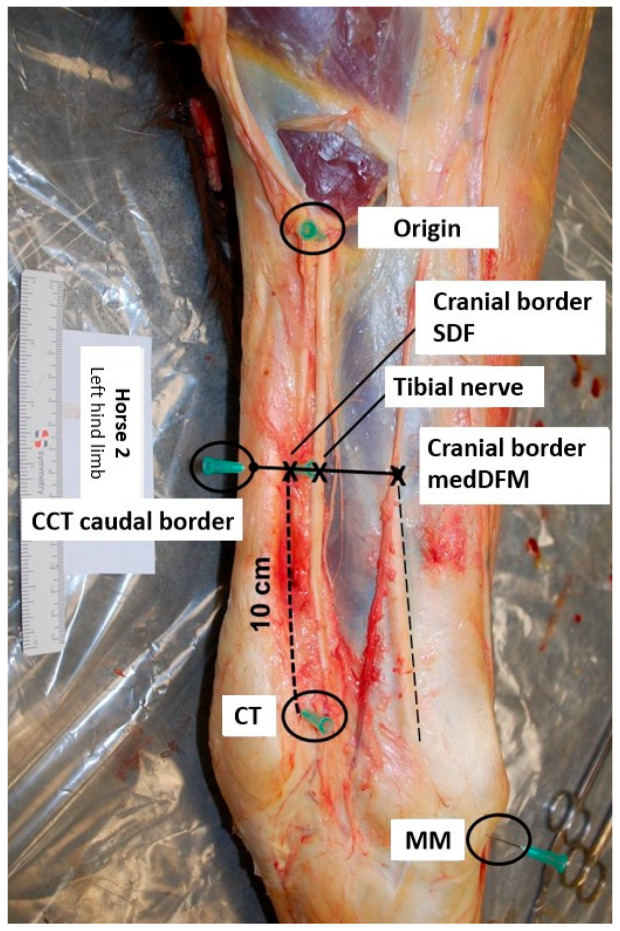
Illustration of the placement of the needles at the anatomical reference points (medial view, top is proximal, left is caudal; CCT: common calcaneal tendon, CT: calcaneal tubercle, MedDFM: medial digital flexor muscle, MM: medial malleolus, SDF: superficial digital flexor).

Anatomical reference points were marked using 21-gauge needles (Figure 1). The first needle was placed at the site where the tibial nerve courses superficially in proximity to the muscle bellies of the gastrocnemius muscle (origin). A second needle was placed at the cranial border of the insertion of the medial collateral ligament of the tarsus to the medial malleolus (MM). Placement of a third needle was just proximal to the calcaneal tubercle (CT). A fourth needle was placed 10 cm proximal to the CT and just cranial to the superficial digital flexor muscle (cranial border SDF). A final needle was placed at the caudal border of the common calcaneal tendon (CCT), at the same height as the fourth needle.

Measurements (mm) were performed using a Stanley Tylon™ 3M (Chiro, Taiwan) measuring tape. The nerve thickness was assessed midway between the origin of the nerve and the branching into the plantar nerves, extending from the dorsal to the plantar aspect, perpendicular to the course of the nerve. The localization of the bifurcation of the tibial nerve into the plantar nerves was measured in relation to the CT and the site of origin of the nerve. The position of the nerve was determined by the distance parallel to the cranial border of the medial digital flexor muscle (MedDFM), the CCT and the cranial border of the SDF. The distances at the mid aspect of the nerve in relation to the cranial border of the MedDFM, the cranial border of the tendon of the superficial digital flexor tendon (SDFT) and the caudal border of the CCT were measured. The first branch originating from the tibial nerve is a fascial branch, which traverses towards the perineural adipose tissue and fascia. The medial cutaneous branch represents a second division of the tibial nerve, running superficially and medially to the tarsus. Measurements starting from the origin of both branches were conducted in relation to the CT, MM and origin.

### 2.3. Data Analysis

For each parameter, the mean, standard deviation (SD), median, minimum, and maximum values were calculated. A Shapiro–Wilk test was used to verify if data presented normal distribution. As data presented significant deviation from a normal distribution, Wilcoxon paired signed rank tests were used to assess differences between paired hind limbs. General calculations were performed using Microsoft Excel (https://www.microsoft.com/en-us/microsoft-365/excel, accessed on 17 July 2024). Statistical analysis was performed using GraphPad Prism 10.

## 3. Results

### 3.1. Anatomical Dissection

**Figure 2:**
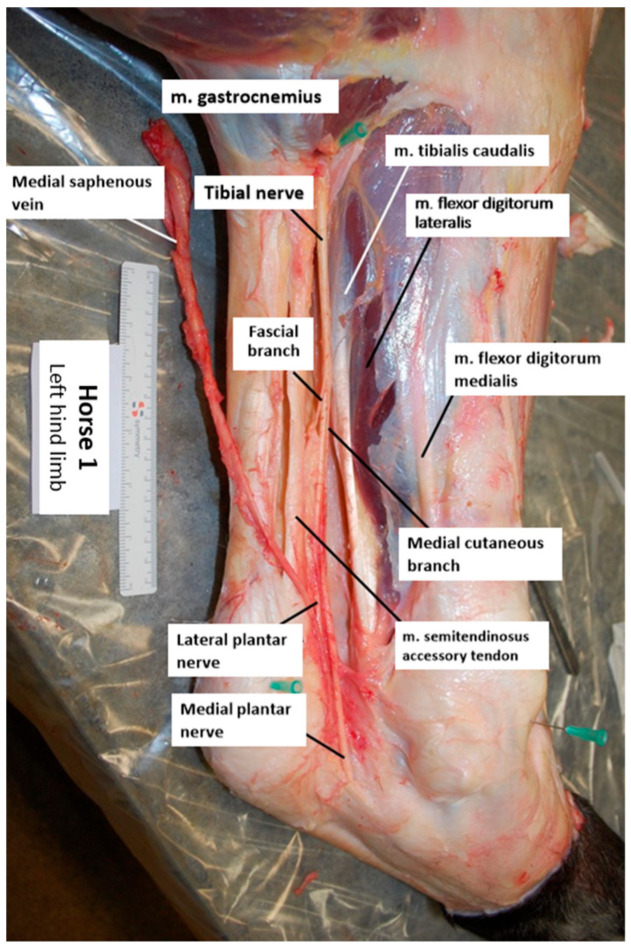
Illustration of a dissected left hind limb (horse 1; medial view, top is proximal, left is caudal).

An example of a dissected specimen is shown in Figure 2. The dissection of the skin along the medial aspect of the limb mostly removed large areas of the fascial layer surrounding the common calcaneal tendon (CCT) and the deep digital flexor tendon (DDFT). In all specimens, the tibial nerve was observed to course superficially along the gastrocnemius muscle on the medial aspect, situated between the CCT and the DDFT. Additionally, the tibial nerve was found to run adjacent to the caudal branch of the medial saphenous vein and the saphenous artery. The fascial branch consistently extended in a plantar distal direction, into the perineural adipose tissue and the surrounding connective tissue enveloping the nerve. The medial cutaneous branch followed a distal course along the medial aspect of the tarsus. A third branch was observed bilaterally in one of the horses. Due to its slender nature, the nerve proved challenging to trace. In the left hind limb of one horse, the nerve was disrupted during dissection, rendering measurements unattainable.

### 3.2. Measurements

Table 1 provides the aggregate statistics, including the mean, median, standard deviation (SD), minimum, and maximum values of all parameters, along with results from paired comparisons between the left and right hind limbs. All measurements are presented in Table 2.

The mean thickness of the tibial nerve was 6 mm ± 1 mm. The nerve bifurcated into the plantar nerves at a maximum height of 85 mm proximal to the CT. The tibial nerve was located at a mean distance of 11 mm ± 5 mm cranial to the border of the SDF. Both branches of the tibial nerve, the fascial branch and medial cutaneous branch, separated at a maximum of 150 mm proximal to the CT. There were no significant differences in any measurements between paired limbs.

## 4. Discussion

This study is the first to investigate if anatomical variations or inadequate external landmarks could explain the lack of efficacy observed during blind perineural anesthesia of the tibial nerve as reported previously [1,6]. Using detailed anatomical dissections on paired cadaver limbs, this study confirmed that the trajectory of the tibial nerve was consistently positioned between the DDFT and the medial aspect of the CCT, which is in agreement with general anatomical description [10]. Additionally, statistical analysis revealed no significant differences between contralateral hindlimbs. Extrapolation of this finding must be performed cautiously, as the current explorative study only involved a relatively small sample of paired limbs. Nevertheless, based on our results, other possible explanations for the commonly observed failure of blind perineural anesthesia of the tibial nerve should be considered.

A first potential reason for inadequate perineural anesthesia could be iatrogenic injection into blood vessels, joints, sheaths or bursae [1]. The tibial nerve is on its craniomedial aspect bordered by the caudal branches of the saphenous vein and artery [10]. Penetration of the vein or artery should easily be distinguished by blood appearing in the needle hub. In this case, the needles trajectory should be adjusted accordingly [5]. Plantar synovial recesses of the tarsocrural joint, tendon sheaths and bursae are located distal to the CT, with the exception of the calcaneal subtendinous bursa (CSB) and the intertendinous bursa (CIB) [11,12]. The CIB and CSB can be seen as a single synovial structure, with the proximal recess extending up to 16.5 cm proximal to the CT [13]. However, accidental injection into this bursa after perineural anesthesia of the tibial nerve has not been documented.

The thickness of the nerve after removal of perineural tissue in this study was 6 ± 1 mm, which is consistent with previous descriptions [4,10]. However, this finding is in contrast with Alexander and Dobson (2003) [14] who documented a nerve thickness of 1.6 mm measured by ultrasonography in a longitudinal plane at the same level. In that study, the nerve was observed to be oval shaped. Consequently, the difference in nerve thickness between studies could be explained by the direction of measurements taken (lateromedial versus dorsoplantar) [14]. It has been described that desensitization of larger nerves takes longer than in smaller nerves and may take up to 20 min or longer [1]. This may be an important factor in the perceived lack of success of desensitization of the tibial nerve.

In the present study, dissection of the tibial nerve posed challenges primarily attributable to the presence of perineural tissue, as well as a fascial layer covering the nerve. In a study regarding perineural injection of the palmar nerves, Nagy et al. (2009) speculated that the presence of anesthetic product outside the fascial sheath enveloping the neurovascular bundle might account for a delay in desensitization [15]. Perineural fat has also been described to act as a barrier for the diffusion of local anesthetic agent [7,8]. Therefore, it seems reasonable to presume that the substantial amount of perineural tissue may also play a role in the challenges associated with achieving desensitization of the tibial nerve.

The bifurcation of the tibial nerve into the plantar nerves was located at a maximum height of 85 mm proximal to the CT. Following the bifurcation of the plantar nerves from the tibial nerve, a proximate course was observed, with both nerves traversing in close proximity for a distance of several centimeters. Based on the results of this study, the distance of 10 cm proximal to the CT described by multiple authors should be adequate for achieving injection proximal to the bifurcation [1,2,3,4,6]. To determine the location of the nerve in craniocaudal direction, the cranial border of the SDF is easily palpated and used as landmark for injection. In this study, the nerve was localized 11 ± 5 mm cranial to the border of the SDF. This parameter was determined on the disarticulated limbs after removal of perineural tissue and fat, and it cannot be excluded that this might have confounded the position of specific anatomical landmarks. A second potential limitation is the difficulty in standardizing limb positioning due to the practical constraints of working with post mortem specimens. This may limit extrapolation to an in vivo situation. In our findings, the tibial nerve was located at a maximum distance of 20 mm cranial to the border of the SDF and coursed along the caudal border of the DDF. Hence, proceeding distally away from the cranial border of the SDF. This implies that the injection should be conducted in closer proximity to the SDF when executed more proximally. The caudal border of the CCT (33 ± 5 mm) could theoretically also be used as landmark, although a landmark as close as possible to the injection site is preferable. The cranial border of the MedDFM (29 ± 4 mm) can only be identified after removal of the fascia, and therefore this does not have any practical value.

The medial cutaneous branch and fascial branch of the tibial nerve both bifurcated at a maximum height of 150 mm proximal to the CT. With the injection located 10 cm proximal to the CT, these branches might not be desensitized despite using large volumes and a fan-shaped injection technique [1,2,3,4]. This will not have an influence on interpretation of orthopedic examinations, since these branches only affect skin sensation. Very proximal debranching could explain why skin innervation is not always effective while deeper structures are desensitized [5]. Wheat and Jones (1981) described separate perineural anesthesia of these branches to obtain complete local anesthesia [6]. In our study, a third branch was found in both limbs of one of the horses, close to the CT (19 mm), and while unfortunately the course of this branch could not be followed, it was most likely one of the cutaneous nerve branches.

Ultrasound-guided perineural anesthesia of the tibial nerve has been described [7]. A cadaveric study and an in vivo study proved the superior accuracy of ultrasound-guided anesthesia in comparison with the blind technique, especially when using a combined cranial and caudal injection to the tibial nerve [8,9]. However, two operators are required for the technique and Bellitto et al. (2024) [9] showed that it takes significantly longer to perform than the blind technique. However, given the 100% success rate of the ultrasound-guided technique, the need for the injection to be repeated would be rare, which would be more time efficient than repeating the blind technique [9]. Practical constraints probably explain why the blind technique is still used in equine practice, but based on the current knowledge, it is recommended to use ultrasound guidance whenever possible to avoid false negative effects of perineural anesthesia of the tibial nerve and consequent delays in the localization of pain causing lameness.

## 5. Conclusions

Based on the anatomical information presented in this study, failure of perineural anesthesia of the tibial nerve cannot be attributed to anatomical variations in the nerve’s course. Our measurements confirm that perineural injection should be performed 10 cm proximal to the proximocranial aspect of the calcaneal tubercle and 11 mm cranial to the border of the superficial digital flexor.

## Figures and Tables

**Table 1:** Reference values of the measurements and statistical comparison between paired hind limbs based on the Wilcoxon paired signed rank test with statistical significance set at *p* < 0.05.

	Mean (SD)	Median (Min.–Max.)	*p*-Value
Nerve thickness (mm)	6 ± 1	6 (5–7)	NS
Bifurcation			
*Plantar nerves* *–CT (mm)*	46 ± 16	41 (25–85)	NS
*Plantar nerves–origin (mm)*	216 ± 25	216 (180–260)	NS
Location of the nerve			
*Cranial border MedDFM (mm)*	29 ± 4	30 (22–33)	NS
*Cranial border SDF (mm)*	11 ± 5	9 (4–20)	NS
*CCT caudal border (mm)*	33 ± 5	33 (25–43)	NS
Fascial branch			
*CT (mm)*	115 ± 16	109 (95–150)	NS
*MM (mm)*	182 ± 17	184 (140–199)	NS
*origin (mm)*	81 ± 14	85 (60–100)	NS
Medial cutaneous branch			
*CT (mm)*	79 ± 41	59 (37–150)	NS
*MM (mm)*	142 ± 28	135 (115–199)	NS
*origin (mm)*	116 ± 30	123 (66–160)	NS
3rd branch			
*CT (mm)*	19 *	19 *	-
*MM (mm)*	95 *	95 *	-
*origin (mm)*	165 *	165 *	-

*: based on one measurement; NS: Not significant (α = 0.05).

**Table 2:** Measurements.

	Horse 1		Horse 2		Horse 3		Horse 4		Horse 5	
	Left Hind	Right Hind	Left Hind	Right Hind	Left Hind	Right Hind	Left Hind	Right Hind	Left Hind	Right Hind
Nerve thickness (mm)	6	5	6	6	7	6	5	5	7	7
Bifurcation										
*Plantar nerves–CT (mm)*	85	40	35	42	38	34	50	25	57	52
*Plantar nerves–origin (mm)*	180	260	225	230	186	198	225	200	250	203
Location of the nerve										
*Cranial border MedDFM (mm)*	30	22	30	33	- *	- *	25	25	32	31
*Cranial border SDF (mm)*	16	20	15	4	- *	- *	5	10	8	6
*CCT caudal border (mm)*	37	43	33	34	- *	- *	25	30	33	32
Fascial branch										
*CT (mm)*	150	- **	125	120	95	109	100	125	101	107
*MM (mm)*	- **	- **	199	199	180	184	140	180	183	190
*origin (mm)*	100	- **	66	65	85	73	85	60	94	97
Medial cutaneous branch										
*CT (mm)*	150	- **	125	49	57	41	60	113	37	- **
*MM (mm)*	- **	- **	199	135	140	123	115	165	115	- **
*origin (mm)*	100	- **	66	137	125	141	120	80	160	- **
3rd branch										
*CT (mm)*					- **	19				
*MM (mm)*					- **	95				
*origin (mm)*					- **	165				

*: hind limbs were too small for this measurement; **: branches were not visible on dissection.

## Data Availability

Data is contained within the article.

## References

[1] Moyer W., Schumacher J., Schumacher J., Solutions A.V. (2011). Regional Anesthesia: Hindlimb Nerve Blocks. Equine Joint Injection and Regional Anesthesia.

[2] Schmotzer W.B., Timm K.I. (1990). Local anesthetic techniques for diagnosis of lameness. Vet. Clin. N. Am. Equine Pract..

[3] Bassage L.H., Ross M.W., Ross M.W., Dyson S.J. (2011). Diagnostic analgesia. Diagnosis and Management of Lameness in the Horse.

[4] Baxter G.M., Stashak T.S., Baxter G.M. (2011). Perineural and Intrasynovial Anesthesia. Adams and Stashak’s Lameness in Horses.

[5] Dyson S. (1984). Nerve blocks and lameness diagnosis in the horse. Practice.

[6] Wheat J.D., Jones K. (1981). Selected techniques of regional anesthesia. Vet. Clin. N. Am. Large Anim. Pract..

[7] Denoix J.M., Beaumont A., Bertoni L. (2020). Ultrasonographic guided block of the tibial nerve. Equine Vet. Educ..

[8] van der Laan M., Raes E., Oosterlinck M. (2021). Cadaveric comparison of the accuracy of ultrasound-guided versus ‘blind’ perineural injection of the tibial nerve in horses. Vet. J..

[9] Bellitto N.A., Voute L., Reardon R., Withers J.M. (2024). Ultrasound-guided perineural injection of the tibial nerve in the horse versus a ‘blind’ technique. Equine Vet. Educ..

[10] Budras K.D., Sack W.O., Röck S. (2011). Pelvic Limb. Anatomy of the Horse.

[11] Raes E.V., Bergman E.H., van der Veen H., Vanderperren K., Van der Vekens E., Saunders J.H. (2011). Comparison of cross-sectional anatomy and computed tomography of the tarsus in horses. Am. J. Vet. Res..

[12] Tomlinson J.E., Redding W.R., Berry C., Smallwood J.E. (2003). Computed tomographic anatomy of the equine tarsus. Vet. Radiol. Ultrasound.

[13] Post E.M., Singer E.R., Clegg P.D. (2007). An anatomic study of the calcaneal bursae in the horse. Vet. Surg..

[14] Alexander K., Dobson H. (2003). Ultrasonography of peripheral nerves in the normal adult horse. Vet. Radiol. Ultrasound..

[15] Nagy A., Bodo G., Dyson S.J., Szabo F., Barr A.R. (2009). Diffusion of contrast medium after perineural injection of the palmar nerves: An in vivo and in vitro study. Equine Vet. J..

